# Variations in Early Response of Grapevine Wood Depending on Wound and Inoculation Combinations with *Phaeoacremonium aleophilum* and *Phaeomoniella chlamydospora*

**DOI:** 10.3389/fpls.2016.00268

**Published:** 2016-03-11

**Authors:** Romain J. G. Pierron, Jérôme Pouzoulet, Christel Couderc, Elodie Judic, Stéphane Compant, Alban Jacques

**Affiliations:** ^1^Equipe Agrophysiologie et Agromolécules, Département des Sciences Agronomiques et Agroalimentaires, Institut National Polytechnique de Toulouse – Ecole d’Ingénieurs de Purpan, Université de ToulouseToulouse, France; ^2^Département BioSym, LGC UMR 5503 (CNRS/UPS/INPT), INP-ENSAT Université de ToulouseCastanet-Tolosan, France; ^3^Department of Botany and Plant Sciences, University of California, RiversideCA, USA; ^4^AIT Austrian Institute of Technology GmbH, Bioresources Unit, Health and Environment DepartmentTulln, Austria

**Keywords:** plant perception, wood gene expression, grapevine, trunk diseases, esca

## Abstract

Defense mechanisms in woody tissue are poorly understood, especially in vine colonized by trunk pathogens. However, several investigations suggest that molecular mechanisms in the central tissue of *Vitis vinifera* L. may be involved in trunk-defense reactions. In this work, the perception of *Phaeoacremonium aleophilum* and *Phaeomoniella chlamydospora* alone or together were investigated in cuttings of Cabernet Sauvignon trunks. Plant responses were analyzed at the tissue level via optical microscopy and at the cellular level via plant-gene expression. The microscopy results revealed that, 6 weeks after pathogen inoculation, newly formed vascular tissue is less developed in plants inoculated with *P. chlamydospora* than in plants inoculated with *P. aleophilum*. Co-inoculation with both pathogens resulted in an intermediate phenotype. Further analysis showed the relative expression of the following grapevine genes: *PAL*, *PR10.3*, *TL*, *TLb*, *Vv17.3*, *STS*, *STS8*, *CWinv, PIN*, *CAM*, *LOX* at 10, 24, 48, and 120 h post-inoculation (hpi). The gene set was induced by wounding before inoculation with the different pathogens, except for the genes *CAM* and *LOX*. This response generated significant noise, but the expression of the grapevine genes (*PAL*, *PR10.3*, *TL*, *TLb*, *Vv17.3*, *STS*, *STS8*, *CWinv*, and *PIN*) still differed due to perception of mycelium by the plant. Furthermore, at 48 hpi, the induction of *PAL* and *STS8* differs depending on the pathogen, and a specific pattern emerges from the different inductions associated with the different treatments. Based on these results, we conclude that *V. vinifera* L. trunk perceives the presence of pathogens differently depending on the inoculated pathogen or even on the combination of co-inoculated pathogens, suggesting a defense orchestration in the perennial organs of woody plants.

## Introduction

Defense mechanisms in woody tissues of trees or vines are still poorly understood. One thing that is known, however, is that defense reactions in woody plants have two components: (i) a wounding response and (ii) other responses that depend on pathogen perception. Since, Hartig’s (1894) work on wood response to injuries, trees are known to heal their wounds by forming bark ridges. On the plant scale, forming defense lines at strategic anatomical locations in the trunk is part of the strategy of compartmentalization of decay in trees (CODIT; Shigo and Marx, 1977). The strength of these lines is critical in trees because it protects the vascular cambium from infected tissues, thus allowing newly functional xylem vessels to regenerate, leading to plant longevity. The trunk of woody plants thus becomes an ecological niche rich in fungal and bacterial species associated with wood decay, as documented for grapevines by Bruez et al. (2012). The function of the barriers described in the CODIT model is to circumvent problems due to cavitation and the spread of pathogens (Pearce, 1996; Smith, 2006). Consequently, in wilt diseases, the xylem appears to be the key tissue because it is where most plant-microbe interactions occur (Yadeta and Thomma, 2013).

The plant response at the cell or tissue scale suggests mechanisms involved in barrier formation at the plant scale. Near injuries, xylem tissues develop reaction zones enriched with lignin and suberin (Hawkins and Boudet, 1996). In axial parenchyma, rays, and vessels, intracellular suberin is deposited near the cell walls (Biggs, 1987; Schmitt et al., 1995; Pearce, 2000; Pouzoulet et al., 2014). These reactions may appear fragmented in the trunk if considered separately, but the association of suberized xylem cells and tyloses in the vessels may shape CODIT barriers at the scale of individual trunks (Biggs, 1987).

The particular lifestyle of vines is responsible for its wood anatomy, which is adapted to its climbing behavior. Consequently, the CODIT model must be applied with care to domesticated vines. For instance, vines present wider vessels than trees, which increases the risk of cavitation (Putz and Mooney, 1991). Vessel width may also explain the susceptibility to tracheiphilous fungi of *Vitis vinifera* L. (Pouzoulet et al., 2014). Nevertheless, some evidence of trait modification upon pathogen attack suggests that molecular mechanisms are active in grapevine-trunk defense. In fact, the accumulation of suberin in pruning wounds correlates with their susceptibility to infection by *Eutypa lata* (Munkvold and Marois, 1995) and may participate in trunk defense more than does the accumulation of lignin (Pouzoulet et al., 2014). Discoloration of wood following the annual growth ring (Mugnai et al., 1999) and the occlusion of xylem vessels (Mori et al., 2001; Pierron et al., 2015a) has also been characterized. More recently, by comparing wounded and inoculated cuttings with wounded and non-inoculated cuttings, Czemmel et al. (2015) revealed that, compared with the latter, the former has a lower starch content 2 months post-inoculation with *Neofusicoccum parvum*. Finally, the formation of reaction zones and wound healing in grapevine is found to vary if a pathogen is present in the wound (Pouzoulet et al., 2013a). This evidence suggests that grapevine-trunk tissues have a certain level of perception to biotic stress—a biological question that, to our knowledge, has not yet been addressed in the context of the CODIT model.

Understanding wound healing and trunk defense against pathogens is particularly important in *V. vinifera*. Grapevine-trunk diseases are known as a worldwide threat to vineyards. The estimates are worrisome: 72% of the plants expressed these diseases and, as a result of these diseases, 12% of French vineyards were unproductive in Grosman and Doublet (2012).

Grapevine trunk hosts several species of fungi, some of them forming a cocktail that is often isolated from the wood of esca-symptomatic plants (Mugnai et al., 1999; Bruez et al., 2014). Esca is one of the main grapevine-trunk diseases, together with Eutypiosis (Bertsch et al., 2013). If fungi associated with esca are isolated in the trunk then, under vineyard conditions, symptoms become visible in the field. The tiger-striped-leaf symptom, also called the grapevine leaf stripe disease (GLSD), is particular to esca (Surico, 2009) and is easy to identify in vineyards in the early summer (Bertsch et al., 2013). Grapevines do not necessarily present foliar symptoms every year (Guerin-Dubrana et al., 2013). Two causes, both highly dependent on environmental factors, can explain the expression of symptoms: (i) fungal toxins secreted by fungi in the trunk and transported to the leaves (Andolfi et al., 2011), and (ii) water stress caused by the disruption of vessels (Lecomte et al., 2012; Pouzoulet et al., 2014). Finally, plants with trunks heavily degraded by fungi may die from apoplexy, which is a sudden wilting of the plant that leads to death (Mugnai et al., 1999).

In cuttings of inoculated grapevine trunk, several pathogen species can cause GSLD symptoms in the wood. For example, species of *Botryosphaeriaceae* were recently found to be pioneers of Botryosphaeria dieback (Úrbez-Torres, 2011; Úrbez-Torres et al., 2014). Another species, *Fomitiporia mediterranea*, is considered a latecomer (Mugnai et al., 1999) and is capable of degrading lignin (Fischer, 2006). *Phaeoacremonium aleophilum* and *Phaeomoniella chlamydospora* are also considered pioneers of young esca (Mugnai et al., 1999; Feliciano et al., 2004; Laveau et al., 2009).

Both *P. aleophilum* and *P. chlamydospora* are often isolated together from grapevine presenting young esca; these lead to the so-called Petri disease (Mugnai et al., 1999). They affect grapevines from 1 to 5 years-old and may cause GLSD. *P. chlamydospora* causes more wood degradation than *P. aleophilum*; however, the two strains are equally aggressive on grapevine cuttings in laboratory conditions (Laveau et al., 2009). These pathogens consist of tracheomycetes, which invade xylem vessels: *P. aleophilum*, for example, was immuno-localized in xylem vessels, fibers, and pith 4 months after internodal inoculation (Fleurat-Lessard et al., 2014), whereas *P. chlamydospora* was identified mainly in xylem vessels and surrounding fibers (Valtaud et al., 2009; Fleurat-Lessard et al., 2010; Mutawila et al., 2011). Both fungi are likely to share the same ecological niche and their synergetic interaction has already been investigated, showing that *P. chlamydospora* secretes toxins affecting the plant and favoring the activity of *P. aleophilum* wood-degrading enzymes (Luini et al., 2010). Nevertheless, recent genomic data predict that these species have a low virulence compared with other plant pathogens (such as *N. parvum*, *Eutypa lata*, or *Fusarium graminearum*), suggesting that they interact with other esca-associated fungi (Blanco-Ulate et al., 2013; Antonielli et al., 2014). Early colonization by *P. aleophilum* 6 and 12 weeks post-inoculation revealed that xylem fibers were colonized prior to xylem vessels and that the plant response varies according to plant tissue. This response was due only to wounding in the internode, but comparison with mock-inoculated plants in the node indicates that the response is particular to the presence of the pathogen (Pierron et al., 2015a). Inoculation with *P. chlamydospora* affects wound healing, which also suggests that grapevine wood may perceive the presence of the pathogen or its effectors (Bruno and Sparapano, 2006; Santos et al., 2006).

Although, trunk defenses have been described as non-specific and as depending only on wounding damage (Blanchette and Biggs, 1992), some recent tissue-scale results from grapevines (Pouzoulet et al., 2013a; Czemmel et al., 2015; Pierron et al., 2015a) and cellular-scale results from non-woody material (Lima and Dias, 2009; Luini et al., 2010; Bénard-Gellon et al., 2014) suggest that a certain level of plant perception contributes to the plant-defense reaction. The altered wound healing in Cabernet Sauvignon cuttings upon inoculation with *P. chlamydospora* (Pouzoulet et al., 2013a) suggests that wood tissues in *V. vinifera* L perceive this pathogen.

The primary aim of the present study was thus to reveal whether plant healing is affected by the presence of the pathogens *P. chlamydospora* or *P. aleophilum* inoculated into a wound or the concomitant presence of both pathogens *P. chlamydospora* + *P. aleophilum* co-inoculated into a single wound. Another aim was to investigate whether woody grapevine tissue is capable of early pathogen perception. Plant response to wounding was monitored on the tissue-scale by comparing symptoms, measuring wound healing, and quantifying fungal DNA in the wood. The early perception of *P. chlamydospora* and *P. aleophilum* was assessed by analyzing the expression of defense-related genes by reverse-transcriptase quantitative polymerase chain reaction (RT-qPCR).

## Materials and Methods

### Fungal Material

*Phaeoacremonium aleophilum* CBS 100398 and *P. chlamydospora* CBS 239.74 were maintained in potato-dextrose agar (PDA, Merck, Germany) in Petri dishes placed in the dark at 26°C. Spore suspensions were inoculated to measure the impact on wound healing. A plug of hyphae from a 3-weeks-old culture was placed in 1 mL of sterilized demineralized water (121°C, 15 min) in a 1.5 mL tube to make a conidia suspension. The tube was then briefly vortexed and centrifuged for 30 s at 2300 *g*. The plug of hyphae was then removed and the tube was centrifuged again for 30 s at 2300 *g* to allow the fungal conidia to precipitate out onto the bottom of the tube. The conidia suspension was then concentrated by pipetting the upper part of the solution to obtain a final volume of 200 μL. The concentration was adjusted by using a counting chamber (Malassez cell) to obtain 20,000 conidia/mL.

### Plant Material

Grapevine material was collected in vineyards and conditioned as described in Pierron et al. (2015a). One-year-old canes of *V. vinifera* L. cv. Cabernet Sauvignon clone 15 were harvested in January 2013 and 2014 (Toulouse, France) and treated with fungicide by soaking canes in 0.05% Cryptonol^®^ for 1 h. Cleaned canes were stored at 4°C until further processing. Canes were divided into cuttings with two dormant buds and cleaned in a 20 L water bath containing 10 mL of bleach (2.5% active chloride) for 1 min before rinsing two times with tap water. Cuttings were then stored at 4°C overnight in an aqueous solution of 0.05% Cryptonol^®^. The plant material was cleaned by three successive washes in baths of sterile tap water and planted in plastic trays filled with moistened autoclaved glass wool. The cuttings were placed in a growing chamber (photoperiod 16/8, 25°C; 90% humidity) and watered with autoclaved tap water. Budding and rooting took 4–6 weeks before cuttings were potted in 7 cm × 7 cm × 8 cm pots containing a sterile mixture of perlite, sand, and turf (1:1:1 v/v). The plants were then transferred to a growth chamber (photoperiod 16/8, 25°C; 45% humidity) and, to avoid potting stress, remained there for at least 1 week before treatments. Following treatment, plants were maintained in the growth chamber (photoperiod 16/8, 25°C; 45% humidity) and watered every other day with autoclaved tap water.

Two different inoculation protocols were used for the microscopy or molecular biology work. Three biological replicates were made for each protocol. Conidia suspensions were injected into a wound to investigate how pathogens affect wound healing 6 weeks post-inoculation. To study the early perception of pathogens by woody tissues, a short time frame was used of 10–120 h post-inoculation (hpi). Within this time frame, the spores barely have enough time to finish their germination, so we chose to inoculate the mycelium plug.

### Microscopy of Wound Healing in *Vitis vinifera* Trunk

#### Inoculation

Plants (*N* = 36, three biological replicates of nine plants) were inoculated when at least six leaves were fully developed. First, plants were partly surface sterilized by wiping with a cloth sprayed with 70% ethanol. A wound was made by mechanical drilling with a 3-mm-diameter, flame-sterilized drill. Three sets of plants were inoculated; the first with 50 μL of a conidia suspension (20,000 conidia/mL) of *P. aleophilum* (*N* = 3 × 3) and the second with an analogous solution of *P. chlamydospora* (*N* = 3 × 3). The third set was co-inoculated with an analogous solution containing both species (*N* = 3 × 3). Mock-inoculated plants (*N* = 3 × 3) were inoculated with sterile water from the same source as used to prepare the conidia suspensions. Plants were sampled 6 weeks post-inoculation.

#### Sampling and Staining

Three-centimeter-long wood samples were harvested near the wound site. The samples were fixed and dehydrated in consecutive ethanol baths (30, 50, and 80%, 30 min each at 4°C; Ruzin, 1999) and then conserved in the final 80% ethanol bath. Samples were rehydrated in ethanol baths of decreasing concentration (50, 30%, H_2_O) and 30 μm sections were cut by using a Leica Vibratome VT 100S with a sapphire DDK knife. Sections were then stained by immersion for 2 min in a filtered aqueous 1% solution of safranin O. After rinsing for 2 min with water, sections were stained by immersion for 1 min in aqueous 1% Astral Blue solution and then rinsed for 1 min with water. Specimens were mounted in glycerol (>95%, Sigma-Aldrich, St. Louis, MO, USA) and sealed with nail polish. Slides were kept at 4°C until observations.

#### Observations

Stained wood sections were mounted on glass slides and observed under a large-field Leica DM IRBE optical microscope (Leica Microsystems CMS GmbH, Wetzlar, Germany). Images were acquired with a Leica 8-bit camera and the Leica Application Suite AF software suite (Leica Microsystems CMS GmbH, Wetzlar, Germany).

### Quantification of Fungal DNA in Wood of *Vitis vinifera*

When at least six leaves were fully developed, plants (*N* = 108, three biological replicates of 36 plants) were inoculated and prepared as described above. They were then inoculated with 50 μL of a conidia suspension (20,000 conidia/mL) of *P. aleophilum* (*N* = 3 × 9) and *P. chlamydospora* (*N* = 3 × 9) and co-inoculated with both species (*N* = 3 × 9). Mock-inoculated plants (*N* = 3 × 9) were inoculated with sterile water from the same source as used to prepare the conidia suspensions. Three plants were sampled at 2, 4, and 6 weeks post-inoculation. A 2-cm-long section of wood was collected around the wounding. One sample consisted of three pooled plants.

We monitored fungal development in grapevine trunk through DNA quantification as per Pouzoulet et al. (2013b). Briefly, 1 mL of extraction buffer (Tris-HCl 100 mM, EDTA 20 mM, NaCl 1.4 M, CTAB 2%, PVPP 2%, β-mercaptoethanol 0.5%, RNAse A 0.4% v/v supply in the DNeasy plant mini kit Qiagen) was added to 100 mg of wood powder contained in a 2 mL tube. The tubes were briefly vortexed, then 500 μL of chloroform-isoamyl-alcohol (24:1) were added, after which the tubes were incubated in ice for 5 min. The mix was centrifuged (2300 *g*, 10 min, 4°C). The supernatant was transferred to a new tube and mixed with AP2 buffer, following which we used the protocol supplied by the DNeasy plant mini kit Qiagen. The DNA concentration was determined by using the qPCR technique with a Quant-it brDNA (Invitrogen) and a fluorimeter Qubit^TM^ (Invitrogen). Reactions proceeded in a final volume of 25 μL, and the reaction mixtures contained 12.5 μL of 2X Plexor Master Mix (Promega). The experiments were done with an ABI 7500 Real-Time PCR cycler (Applied BioSystems, Foster City, CA, USA) equipped with ABI SDS software v.1.4 (Applied BioSystems, Foster City, CA, USA) with the default configuration. The cycling program consisted of (1) an initial denaturation step at 95°C for 5 min, (2) 40 cycles of 5 s at 95°C (for denaturation) followed by 35 s at 65°C (for both annealing and extension), and (3) an additional melting analysis of 40 min from 60 to 95°C. Plexor^TM^ Analysis Software 1.5.6.2 (Promega) was used to analyze the data. Labeled Plexor^TM^ primers (5′Me-iso-dC) were synthesized by Eurogentec S.A. PchQR: 5′-(6-carboxytetra-methylrhodamine) TAMRA labeled (CCATTGTAGCTGTTCCAAAGATCAG); PalQF: 5′-(6-carboxyl-X-rhodamine)ROX labeled (CGGTGGGGTTTTTACGTCTACAG; Liege Science Park, Seraing, Belgium). Unlabeled primers (PchQF: CTCTGGTGTGTAAGTTCAATCGACTC; PalQR: CGTCATCCAAGATGCCGAATAAAG) were synthesized by Invitrogen (Fisher Bioblock Scientific, Illkirch, France).

### Analysis of Expression of Defense-Related Genes in *Vitis vinifera* Wood

#### Inoculation

In this experiment, 3-mm-long, 1-mm-diameter cylindrical plugs of mycelium grown in PDA were injected with a 5 mL syringe into wounds in plants (*N* = 255). To avoid selecting fungal material at differing reproductive stages or with differing cell activity, only hyphae from the periphery of the growing fungi were collected. The plants were therefore inoculated with mycelium of *P. aleophilum* (*N* = 60) and of *P. chlamydospora* (*N* = 60) and co-inoculated with both strains (*N* = 60) or with sterile PDA medium (*N* = 60), and the inoculated wound was covered with cellophane. Another set of plants was left unaltered (*N* = 15).

At 10, 24, 48, and 120 hpi, the *N* = 60 plants per treatment were harvested, which represent *N* = 15 plants per kinetic point per treatment. With a flamed pruning shear, trunk sections were taken from as close as possible to the inoculation sites. For molecular biology, the samples consisted of (*N*′ = 3) samples of *N* = 5 pooled samples per kinetic point per treatment. The unaltered control set consisted of *N* = 15 samples harvested at 120 hpi, because we assumed that the constitutive induction factor (IF) of defense-related genes varies negligibly on the scale of hours. A total of *N*′ = 51 samples were immediately put into liquid nitrogen and stored at -80°C before RNA extraction.

#### cDNA Synthesis for RT-qPCR Analyses

For RNA extraction, samples were ground in liquid nitrogen by using a Retsch MM300 mortar grinder (60 s, 25 oscillations per second, two cycles; Retsch, Germany) in a 35 mL stainless-steel grinding jar (Retsch, Germany) with 20 mm stainless-steel balls (Retsch, Germany). The subsequent protocol was adapted from Southerton (1998) and follows Pierron et al. (2015b). The wood powder (100–200 mg) was incubated 10 min at 65°C in a RNA-extraction buffer (CTAB 2%, PVPP 2%, Tris 300 mM, EDTA 25 mM, NaCl 2 M, pH = 8, β-mercaptoethanol 2%) and centrifuged (15 min, 10,000 rpm, 4°C). The liquid phase was carefully transferred into a new 2 mL tube. One volume of a phenol-chloroform-isoamyl alcohol solution (25:24:1) was added (between the different steps of this protocol, the samples were kept on ice to the extent possible). Next, the mixture was centrifuged (30 s, 10,000 rpm, 4°C) and the supernatant was mixed with one volume of chloroform-isoamyl alcohol solution (24:1) and centrifuged. This cleaning step was repeated and the supernatant was transferred into a new tube with one half volume of 8 M LiCl solution. The samples were then stored overnight at -80°C. The next day the samples were centrifuged (30 min; 10,000 rpm, 4°C) until a pellet appeared at the bottom of the tube. The pellet was dissolved in 250 μL of SSTE buffer (SDS 0.5%, NaCl 1 M, Tris 10 mM, EDTA 1 mM, pH = 8.0), mixed with two volumes of absolute ethanol and then transferred into a Qiagen cleaning column supplied by the manufacturer (RNeasy plant mini Kit, Qiagen, USA). The subsequent steps used the buffers, materials, and protocol supplied with the RNeasy plant mini Kit. The final elution volume was 50 μL and the samples were stored at -80°C.

Early plant responses to stress was assessed by measuring mRNA expression of defense-related genes in tissue surrounding the wounds. Complementary DNA had to be generated from RNA samples prior to analysis by RT-qPCR. Total RNA was quantified by measuring the optical density with a Biophotometer Plus (Eppendorf AG, Germany). A DNase reaction (1 U/l.30 min at 37°C, DNase I, RNase free kit, Fermentas, Canada) was used to ensure that no contaminating genomic DNA was present, and the result was verified to be DNA-free by a PCR that used the total RNA extract as a template and primers of the reference gene Elongation Factor 1 alpha (*EF1α*, see **Table 1**). DNA-free RNA was used for cDNA synthesis using the Maxima First strand cDNA synthesis kit for RT-PCR (Frementas, Canada), starting from 1 μg of total RNA. qPCR experiments were conducted with an ABI 7500 Real-Time PCR cycler (Applied BioSystems, USA) equipped with ABI SDS software v.1.4 with the default configuration. The cycling program consisted of (i) denaturation at 50°C for 2 min and then at 95°C for 10 min, (ii) 40 cycles of 15 s at 95°C for denaturation, followed by 1 min at 60°C for both annealing and extension, and (iii) an additional melting analysis consisting of 40 min final denaturation from 60 to 95°C. The 2^-ΔΔCt^ method from Livak and Schmittgen (2001) was used to calculate the gene expression relative to the housekeeping gene *EF1α*. As discussed in detail in Section “Results,” we selected a set of 11 defense-pathway gene markers. **Table 1** lists their functions and primer sequences.

**Table 1:** Primers and functions of the selected gene sets.

Gene	Sequence	Function	Reference
*EF1-α*	F : GAACTGGGTGCTTGATAGGC	Elongation Factor 1α: reference gene.	Terrier et al., 2005; Borges et al., 2014
	R : AACCAAAATATCCGGAGTAAAAGA		
*LOX9*	F : CCCTTCTTGGCATCTCCCTTA	Lipoxygenase: oxidize lipids in hydroperoxide derivatives. Some are involved in jasmonic acid (JA) synthesis.	Turner et al., 2002; Aziz et al., 2003
	R : TGTTGTGTCCAGGGTCCATTC		
*PAL*	F : TGCTGACTGGTGAAAAGGTG	Phenylalanine ammonia-lyase: key enzyme in phenylpropanoid skeleton synthesis (lignins, flavonoids, and coumarins pathway). PAL is a marker of salicylic acid pathway (SA).	Jones, 1984; Aziz et al., 2003
	R : CGTTCCAAGCACTGAGACAA		
*PR10.3*	F : CGTTAAGGGCGGCAAAGAG	Ribonucleolytic activity. Might be involved in plant response to virus infection. SA marker gene.	Castro et al., 2008
	R : GCATCAGGGTGTGCCAAGA		
*TLb*	F : CTGGAGATGTATGGAACTGATAGTG	Thaumatin-like: PR5 protein presenting antifungal properties.	Perazzolli et al., 2010
	R : TCGGATTTTGAAGACCCTTTAC		
*TL*	F : CCTAACACCTTAGCCGAATTCGC		Spagnolo et al., 2011
	R : GGCCATAGGCACATTAAATCCATC		
*CAM*	F : TATTCCAGTAGTTTGGGTTGGTAGTG	Calmodulin: enzyme involved in cascade signaling during plant-microbe perception. Sensor proteins of cytosolic Ca^2+^ flux.	Perazzolli et al., 2010
	R : AAGAAGCACCAAACAAGAAAGGAG		
*STS8*	F : AAGACATGTGTTGAGTGAGTATGGTA	Stilbene synthases: catalyze 3 malonyl CoA and a coumaryl CoA condensation to form a resveratrol (diphenyl molecule that can form several antifungal compounds such as viniferins, pterostilbenes orpiceids.	Robert et al., 2001; Dai et al., 2012
	R : CTCGATGGTCAAGCCTGGT		
*STS*	F : GTGGGGCTCACCTTTCATT		Robert et al., 2001
	R : CTGGGTGAGCAATCCAAAAT		
*CWinv*	F : ACATTGGCTATTGACGGTGAA	Cell-wall invertase: cell-wall enzyme hydrolyzing apoplastic sugars for cell supplying in energy. Inform about the sink strength of the considered organ.	Sherson et al., 2003; Polesani et al., 2010
	R : ACTCACAACTCTACATACATCT		
*Vv17.3*	F : GTACCATCAGACCACCCATAAGTAGTG	Unknown function. SA marker gene.	Bordiec et al., 2011
	R : AGACCAACGGCAAATCAAGTG		
*PIN*	F : GCAGAAACCATTAAGAGGGAGA	Proteinase inhibitor.	Ryan, 1978; Farmer and Ryan, 1992; Belhadj et al., 2008
	R : TCTATCCGATGGTAGGGACACT		


### Data Analysis

The data were analyzed by using the R software (R Development Core Team, 2013). Contrary to data from healing tissues, data for analyzing gene expression were linearized by natural logarithm transformation. The data were plotted on a *q-q* plot to verify normality, and homogeneity of variance was assessed by using Levene’s test. Having these two conditions satisfied allowed us to use a general linear model to statistically confirm whether the treatments differ significantly from each other with an error α = 0.05. Treatments that differed significantly were separated by using the Tukey *post hoc* test.

## Results

### Effect of Co-inoculation with *P. aleophilum* and *P. chlamydospora* on Plant Healing and Fungal Colonization

**FIGURE 1:**
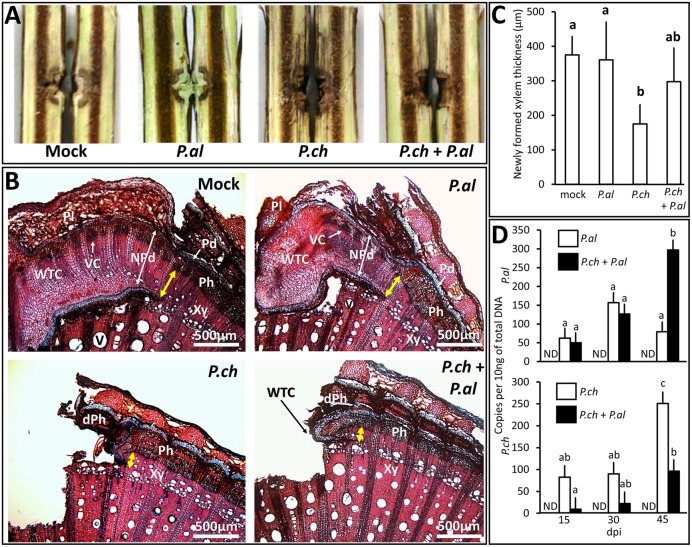
**Impact on wound healing of *P. aleophilum* and *P. chlamydospora* and their growth in *Vitis vinifera* wood.** **(A)** Plants injured and inoculated with *P. aleophilum* alone (*P. al*), *P. chlamydospora* alone (*P. ch*) or both species (*P. al* + *P. ch*). **(B)** Cross section of cv. Cabernet Sauvignon wood taken from near the inoculation site. Plants were injured and inoculated with sterile PDA, *P. aleophilum*, *P. chlamydospora*, and *P. aleophilum* + *P. chlamydospora*. The yellow arrows indicate the thickness of newly formed xylem (yellow bars = 500 μm). dPh is for dead phloem; NPd is for necrophylactic periderm; Pd is for periderm; Ph is for phloem; Pl is for Phellem; V is for vessel; Vc is for vascular cambium; WTC is for wood tissue closure; Xy is for xylem. **(C)** The thickness of healing tissue was measured for the various treatments and the averages were compared by using an analysis of variance test followed by a Tukey test. The letters indicate statistical differences between treatments with an error α = 0.05. **(D)** Fungal development assessed by using qPCR Plexor^TM^, which counts the number of DNA copies per 10 ng of total DNA. Each graph quantifies a pathogen species according to whether it was inoculated alone in the trunk (white bars) or co-inoculated (black bars). ND means non-detected. Results were compared by using an analysis of variance test followed by a Tukey test.

Plant inoculation with *P. aleophilum* and *P. chlamydospora* required a mechanical injury by drilling that induced a response by plant tissue, as observed in mock-inoculated plants at 6 weeks post-treatment (**Figure 1A**). For plants opened longitudinally, the macroscopic phenotype observed in mock-inoculated plants consisted of a thin layer of xylem presenting a brown discoloration around the wound. These tissues were covered by a whitish bark ridge that filled nearly the entire wound. Plants inoculated with *P. aleophilum* (**Figure 1A**) presented the same phenotype as the mock-inoculated plants. Plants inoculated with *P. chlamydospora* did not develop scar tissue over the wound (**Figure 1A**). In addition, tissues adjacent to the inoculation site developed dark discolorations in the longitudinal section in the form of stripes. In plants co-inoculated with both *P. aleophilum* and *P. chlamydospora*, no scar tissue covered the wound. However, an intermediate phenotype appeared in xylem tissue, as revealed by xylem discolorations that were less severe than for plants inoculated with *P. chlamydospora* alone (**Figure 1A**).

A more precise determination of how treatment affected plant healing required further investigations (see **Figures 1B–D**). **Figure 1A** illustrates how scar tissue formed on the damaged vascular cambium. This scar tissue completely covered the injury in plants that were wounded and inoculated with sterile PDA. Pouzoulet et al. (2013a) has already described the tissues present in such grapevine-bark ridges, so no further details are presented here. In plants inoculated with *P. chlamydospora* (**Figure 1B**), scar tissues initiated (note presence of necrophylactic periderm and callus) but failed to develop. In plants inoculated with *P. aleophilum* (**Figure 1B**), the amount of and the histological organization of the bark ridge seemed to be the same as for the mock-inoculated plants. Interestingly, plants co-inoculated with both species (**Figure 1B**) presented an intermediate state where the development of scar tissues was partially restored compared with plants inoculated with *P. chlamydospora* alone (**Figure 1B**). The thickness of the newly formed xylem (**Figure 1C**) showed that plants inoculated with *P. chlamydospora* developed a significantly smaller amount of vascular tissues than mock- and *P. aleophilum*-inoculated plants. In fact, the co-inoculation treatment showed an intermediate phenotype, because the thickness of the newly formed xylem did not differ significantly from that obtained with the other treatments (**Figure 1C**).

To determine *in planta* how the presence of *P. aleophilum* may affect the growth of *P. chlamydospora* (and vice versa), we used qPCR to assess the colonization of *P. aleophilum* and *P. chlamydospora* at 6 weeks post-treatment (**Figure 1D**). Compared with the single-inoculation treatments, significantly less *P. chlamydospora* and more *P. aleophilum* DNA appeared at 45 dpi after co-inoculation (**Figure 1D**).

### Analysis of Defense-Related-Gene Expression in *Vitis vinifera* Wood

#### Selection of Gene Set

Defense-related genes that respond to fungal inoculation in grapevine leaves were selected to cover cell signaling, jasmonic- and salicylic-acid pathways, and genes coding for particular enzymes involved in plant defense. *TL* and *EF1α* genes have already been studied in grapevine woody tissue. Finally, although *CWinv* belongs to the primary metabolism, sugar accumulation may vary in storage organs as a function of the plant defense activation. Gene functions, sequences, and the related publications are listed in **Table 1**.

#### Wood Perception to Wounding in *Vitis vinifera*

Wounding caused significant stress, requiring a separate investigation of the grapevine-trunk response. Relative gene inductions in mock-inoculated plants were obtained by using the 2^-△△Ct^ method, in contrast to the expression in untreated individuals (**Supplementary Figure S1**). In this case, IFs were considered biologically significant when 0.5*x* < IF > 2*x*, as done in Spagnolo et al. (2011), and were linearized by plotting on a log2 scale. The internode intensely responded to injuries 10–120 h post-treatment. Of the eleven genes selected for this study, the expression of seven genes were up-regulated (*PAL*, *PR10.3*, *TL*, *TLb*, *Vv17.3*, *STS*, and STS8) by wounding, the expression of two genes were unaffected (*CWinv* and *PIN*), and the expression of the two remaining genes were repressed at certain kinetic points (*CAM* was repressed at 10 and 120 hpi, and *LOX* was repressed at 24, 48, and 120 hpi compared with relative inductions measured in untreated plants).

#### Perception of Pathogen by Injured Wood

**FIGURE 2:**
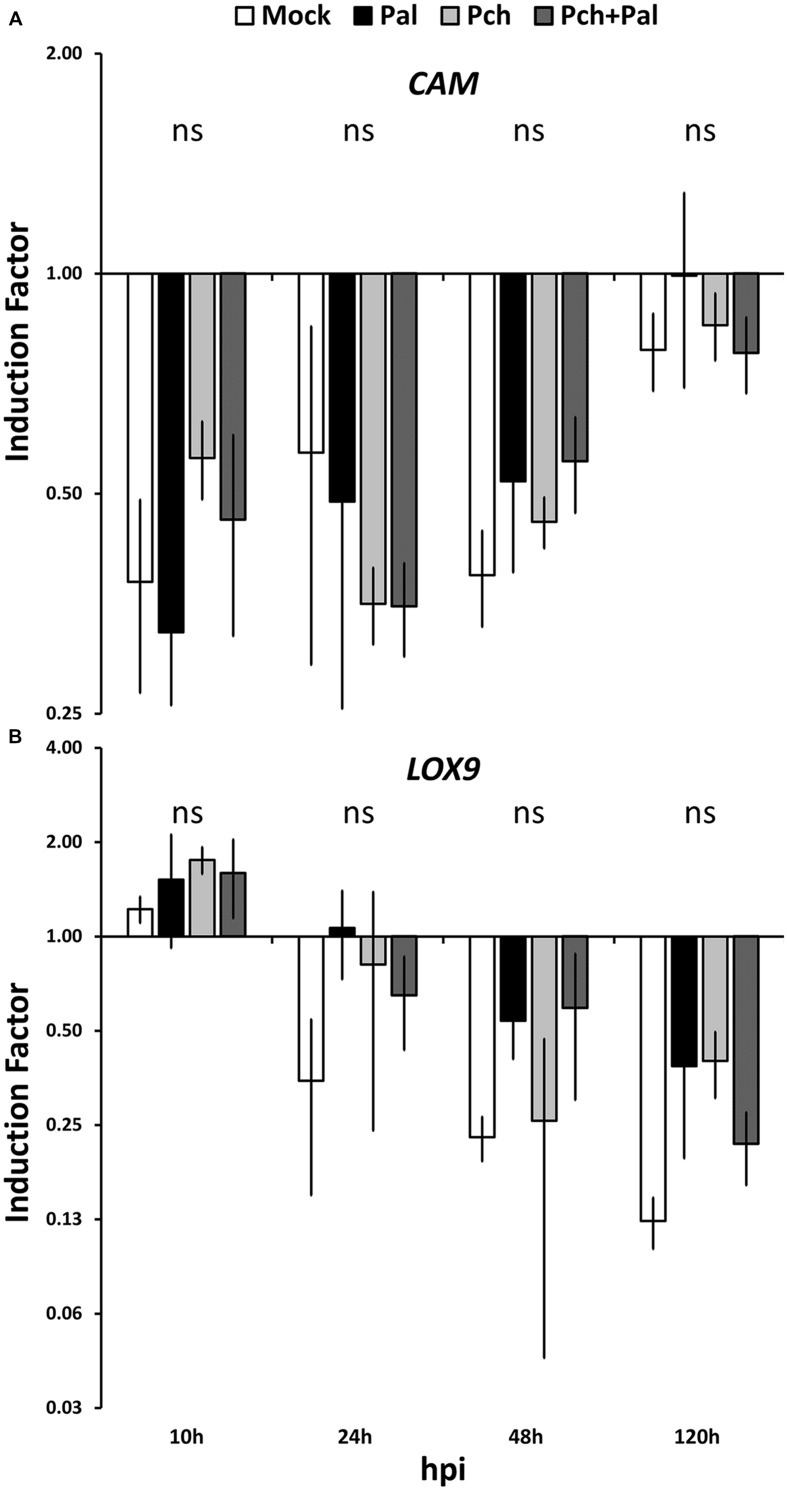
**Short-term kinetics of gene expression in wood of *V. vinifera* L. cv. Cabernet Sauvignon clone 15.** The kinetics of gene expression for the defense-related genes *CAM* **(A)** and *LOX9* **(B)** were acquired 10, 24, 48, and 120 h post-inoculation. Plants were wounded and inoculated with an agar plug for mock inoculation, with *P. aleophilum* (*P. al*), *P. chlamydospora* (*P. ch*) separately for single inoculation, and with both *P. aleophilum* and *P. chlamydospora* for co-inoculation (*P. al* + *P. ch*). Induction factors are relative to the expression of the reference gene *EF1*-α in both the inoculated wood and in the mock-inoculated wood. Error bars correspond to ±1 standard deviations from the mean IF obtained from three biological replicates. The data were analyzed by using an analysis of variance test followed by a Tukey *post hoc* test. The letters indicate statistical differences between treatments with an error α = 0.05 (“ns” means “not significant”).

**FIGURE 3:**
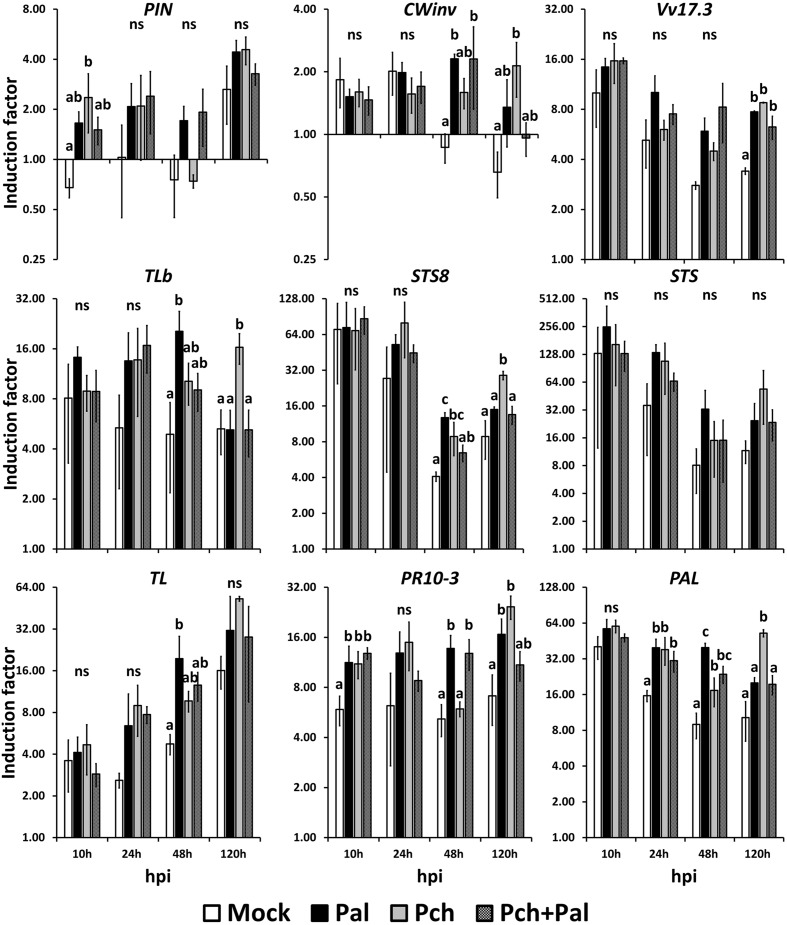
**Short-term kinetics of gene expression in wood of *V. vinifera* L. cv. Cabernet Sauvignon clone 15.** The kinetics of gene expression for the defense-related genes *PIN*, *CWinv*, *Vv17.3*, *TLb*, *STS8*, *STS*, *TL*, *PR10.3*, and *PAL* were acquired at 10, 24, 48, and 120 h post-inoculation. Plants were wounded and inoculated with an agar plug for the mock inoculation, with *P. aleophilum* (*P. al*) and *P. chlamydospora* (*P. ch*) separately for single inoculation, and with both *P. aleophilum* and *P. chlamydospora* for co-inoculation (*P. al* + *P. ch*). Induction factors are relative to the expression of the reference gene *EF1*-α in both the inoculated wood and in the mock-inoculated wood. Error bars correspond to ±1 standard deviation from the mean induction factor obtained from three biological replicates. The data were analyzed by using an analysis of variance test followed by a Tukey *post hoc* test. The letters indicate statistical differences between treatments with an error α = 0.05 (“ns” means “not significant”).

We assessed the trunk response to pathogens in a wound. In this case, the relative inductions resulting from different treatments must be compared, which requires statistically discriminating between gene expressions. The expressions of the genes *CAM* and *LOX9* were not affected by any treatments used in this study (see **Figure 2**). The expressions of the nine remaining genes studied were strongly modified by fungal inoculation in the wood (see **Figure 3**).

Compared with mock-inoculated plants, inoculation by *P. chlamydospora* caused a two-fold induction of *PIN* at 10 hpi (*F* = 6.086, *p*-value = 0.0231 < 0.05; see **Figure 3**). Again compared with mock-inoculated plants, the expression of the gene *CWinv* was up-regulated both in plants inoculated with *P. aleophilum* and in those co-inoculated with *P. aleophilum* + *P. chlamydospora* at 48 hpi (*F* = 6.904, *p*-value = 0.0131 < 0.05; see **Figure 3**). At 120 hpi, the expression of *CWinv* was also up-regulated in plants inoculated with *P. chlamydospora* compared with mock-inoculated plants (*F* = 6.564, *p*-value = 0.015 < 0.05; see **Figure 3**). However, compared with the expression measured in the untreated plants, the expression of these two genes was induced fourfold by the different treatments. Thus, we considered these genes to be weakly up-regulated by the presence of a pathogen in the wood tissue.

At earlier time points, the expression of the gene *Vv17.3* was high in all treated tissues and decreased over time. However, the transcript level remained higher in the fungus-infected tissues than in the mock-inoculated tissues. Compared with the mock-inoculated plants, mycelium injection caused a twofold induction at 120 hpi (*F* = 25.39, *p*-value = 0.00459 < 0.05; see **Figure 3**). At 10 hpi, the expression of *TLb* increased in fungus-treated tissues compared with mock-inoculated tissues; at 48 and 120 hpi, the expression of this gene depended on the fungal species. In fact, at 48 hpi, *TLb* was up-regulated fourfold in *P. aleophilum*-inoculated plants with respect to mock-inoculated plants (*F* = 5.359, *p*-value 0.0257 = < 0.05). In addition, at 120 hpi, the expression of this gene in *P. chlamydospora*-inoculated plants was also up-regulated fourfold with respect to mock-inoculated plants (*F* = 7.976, *p*-value = 0.00867 < 0.05; see **Figure 3**).

For all treatments, the expression of *STS8* and *STS* was high, with the transcript levels obtained at 48 hpi seeming to increase at 120 hpi. To be more specific, starting at 24 hpi, gene induction was higher in fungus-infected tissues than in mock-inoculated tissues, although statistically significant differences occurred only for *STS8*. At 48 hpi, this gene was induced threefold more in *P. aleophilum*-inoculated plants and twofold more in *P. chlamydospora*-inoculated plants than in mock-inoculated plants (*F* = 14.37, *p*-value = 0.00224 < 0.05). At 120 hpi, expression of *STS8* was induced fourfold more in *P. chlamydospora*-inoculated plants than in mock-inoculated plants (*F* = 13.59, *p*-value = 0.00166 < 0.05; see **Figure 3**).

The transcript levels of the gene *TL* increased linearly from 10 to 120 hpi. At 10 hpi, tissues responded similarly to the treatments, whereas *TL* was induced more in fungus-treated tissues than in mock-inoculated tissues at later time points. The transcript levels of this gene also depended on the pathogen species (at 48 hpi for *P. aleophilum* and at 120 hpi for *P. chlamydospora*); however, only the expression of *TL* in *P. aleophilum*-inoculated plants was statistically different than that of the mock-inoculated plants. In fact, *TL* was induced fourfold more in *P. aleophilum*-inoculated plants that in mock-inoculated plants at 48 hpi (*F* = 5.359, *p*-value = 0.0257 < 0.05; see **Figure 3**).

Compared with untreated tissue, the transcript level of *PR10.3* attained high levels within hours of inoculations and remains constant at these levels. At 10 hpi, the presence of mycelium in plants inoculated with *P. aleophilum*, *P. chlamydospora*, or *P. aleophilum + P. chlamydospora* induced expression of *PR10.3* twofold more than in mock-inoculated plants (*F* = 6.473, *p*-value = 0.0156 < 0.05). At 24 hpi, expression of *PR10.3* did not differ between the various treatments. Inoculation with *P. aleophilum* and *P. aleophilum + P. chlamydospora* induced the expression of this gene twofold more than in mock-inoculated plants at 48 hpi (*F* = 13.57, *p*-value = 0.00167 < 0.05). Expression of *PR10.3* at 120 hpi in *P. aleophilum*- or *P. chlamydospora*-inoculated plant tissues was induced respectively two- and fourfold more than in the mock-inoculated plant tissue (**Figure 3**).

Compared with the uninjured control plants, the transcript level of the gene *PAL* was high in all treated tissues; however, it decreased slightly over time. *PAL* was up-regulated by the presence of mycelium at 24 (*F* = 8.663, *p*-value = 0.0068 < 0.05), 48, and 120 hpi compared with uninfected tissues, and the IF depended on the pathogen species for this gene at 48 and 120 hpi. *P. aleophilum* infection induced this gene twofold more than *P. chlamydospora* infection and fourfold more than in mock-inoculated tissues at 48 hpi (*F* = 20.23, *p*-value = 0.00431 < 0.05). *P. chlamydospora* inoculation induced *PAL* expression sixfold more than mock-inoculated plants at 120 hpi (*F* = 9.122, *p*-value = 0.00583 < 0.05; see **Figure 3**).

Thus, for nine of eleven genes, the presence of mycelium (or mycelia) led to different IFs compared with mock-inoculated plants. This result suggests that grapevine trunk may perceive *P. aleophilum* and *P. chlamydospora* differently. For the two non-conforming genes *PAL* and *STS8*, the IFs depended on the pathogen. For *STS8*, the IF caused by *P. aleophilum* exceeded that caused by *P. chlamydospora*, and the latter also differed from the IF caused by *P. aleophilum*+ *P. chlamydospora* and from the IF obtained for the mock-inoculated plants (**Figure 3**, 48 hpi). Compared with the presence of *P. chlamydospora* in the injury, the presence of *P. aleophilum* doubled the IF for *PAL*, and the IFs of both *P. chlamydospora* and *P. aleophilum* exceeded that obtained from mock-inoculated plants (**Figure 3**, 48 hpi). The pattern of gene induction for the other nine genes suggested that grapevine wood perceived these pathogens differently. In fact, at various kinetic points, *P. aleophilum* and *P. chlamydospora* up-regulated gene expression in the plant with respect to the mock-inoculated plants. By listing the genes that were induced at the various kinetic points, an expression pattern due to *P. aleophilum* or *P. chlamydospora* emerged. The most significant pattern indicated the perception of *P. aleophilum* at 48 hpi and of *P. chlamydospora* at 120 hpi. This pattern appeared in the relative expression of the genes *CWinv*, *TLb*, *STS8*, *PR10.3*, *TL*, and *PAL*.

## Discussion

The increasing number of emerging diseases in woody plants such as GTDs makes it vital to understand the defense mechanisms deployed in woody tissues against wounding and pathogens. The present study investigates the response of grapevine woody tissue to wounding and fungal infection at both the microscopic and molecular scale. In addition, we investigate the effect of the co-inoculation by two pathogens sharing the same niche but presenting different colonization strategies.

Because the fungus *P. aleophilum* is reported to inhibit callus growth (Bruno and Sparapano, 2006), the first objective of this study was to assess at the histological level how infection by *P. aleophilum* may affect wound healing. In contrast to *P. chlamydospora*, the results indicate that *P. aleophilum* infection does not significantly affect the healing process or cause streaking. These conclusions are consistent with previous studies that found that *P. aleophilum* isolates cause a weak macroscopic pathogenic effect in woody tissue (Laveau et al., 2009; Luini et al., 2010). However, as observed by Pouzoulet et al. (2013b) and by Pierron et al. (2015a), the tissue can be successfully colonized by *P. aleophilum* without displaying the symptoms. This result may be explained as resulting from the secretion by this fungus of poorly virulent effectors (Luini et al., 2010). By comparing with mock-inoculated plants, the results show that the response of grapevine wood to microbes inoculated *via* wounds depends on the pathogen species. This result suggests that each fungus interacts differently with grapevine xylem tissue.

This conclusion is supported by the results of optical microscopy. As previously reported, the development of callus and healing tissue is reduced upon inoculation with *P. chlamydospora* (Santos et al., 2005; Díaz et al., 2009; Pouzoulet et al., 2013a). Wound healing is partially restored when both fungi are co-inoculated into the same plant. Less *P. chlamydospora* DNA is found in co-inoculated tissue than in single-inoculated tissue. We thus hypothesize that this reduced colonization is associated with a lower pathogenic effect and a slightly higher production of healing tissue.

Previous studies looking at the colonization strategies of *P. chlamydospora* and *P. aleophilum* led to the hypothesis that co-inoculation by these pathogens might result in a more aggressive pathosystem (Graniti et al., 2001; Sparapano et al., 2001). This may be possible if a synergy develops whereby *P. chlamydospora* reduces the defenses of the host by secreting virulent effectors while *P. aleophilum* alters the integrity of the cell walls (Valtaud et al., 2009; Luini et al., 2010). However, if we consider the development of streaking and healing tissue, such a synergy between fungi is not necessarily true *in planta*, at least in the short term. Interestingly, Sparapano et al. (2001) also reported a mutual spatial exclusion of these pathogens in cross-inoculated vines. Here, the decrease in the colonization rate of *P. chlamydospora* in co-infected tissue is also associated with an increase in the colonization rate of *P. aleophilum*. The long-term effect of the increased colonization rate of *P. aleophilum* upon co-inoculation with *P. chlamydospora* should be further investigated.

The observed differences in colonization rates of *P. chlamydospora* and *P. aleophilum* upon co-inoculation could be due to a combination of mechanisms that are not mutually exclusive. First, a modified growth rate due to a direct interaction between *P. aleophilum* and *P. chlamydospora* cannot be excluded. Second, because *P. aleophilum* is a more efficient wood degrader than *P. chlamydospora*, a competition for resources may also occur in host tissues (Santos et al., 2005; Valtaud et al., 2009; Morales-Cruz et al., 2015). Third, modifications in the host response due to the actions of pathogen effectors and a variation in the perception of the pathogens might also affect the ability of *P. aleophilum* and *P. chlamydospora* to colonize their host.

The possible perception by grapevine trunk of fungi suggests the existence of inducible defenses in woody tissues. Active defenses in the trunk are still under debate because modifications of water and oxygen content in the trunk microenvironment caused by the wound may be the cause of the macroscopic symptoms observed in the field (Pearce, 2000). However, the variation of macroscopically observed wood symptoms and the wound healing observed microscopically suggests a phenotypic plasticity in these tissues, which results from the plant capacity to perceive and provide a response to unpredictable environmental stress (Karban and Baldwin, 1997). This plant response requires *de novo* protein synthesis, and thus mRNA transcription. Our goal in this study is to investigate fungal perception by grapevine trunk tissues by analyzing the short-term expression of genes related to plant defense in grapevines.

At 10 hpi and later, the expression of *PAL* was already affected by the wound. This gene is a switch between the primary and the secondary metabolisms; more precisely, it embodies the phenylpropanoid pathway. The genes commonly associated with this pathway (*PR10.3*, *STS*, *STS8*, *Vv17.3*) were also up-regulated early in the kinetics, indicating that a rapid signal occurs (i.e., within hours of infection) in grapevine woody tissue. Surprisingly, expression of the *LOX9* gene was not affected by any treatment in this study, whereas a *LOX* gene is known to be induced by wounding in *Arabidopsis* (Turner et al., 2002). However, within the time frame considered in this work, this particular *LOX* gene may not be a marker of wounding stress in the trunk of *V. vinifera*.

The response to wounding masked any effect of pathogen perception on gene induction at 10 and 24 hpi. At 10 hpi, only the genes *PIN* and *PR10.3* were up-regulated by the presence of mycelium compared with the mock-inoculated plants. Nevertheless, this effect disappeared at 24 hpi, and it remains questionable whether this early perception of mycelium in the trunk (within 10 hpi) was biologically significant. Despite the noise associated with the wounding, the perception by the trunk of mycelium was observed for nine out of eleven genes. More interestingly, for the two genes *STS8* and *PAL*, the trunk responded differently at 48 hpi when *P. chlamydospora* or *P. aleophilum* was inoculated into the injury. To the best of our knowledge, this constitutes the first evidence of an early response specific to biotic stress in the wood of *V. vinifera* L.

Upon comparing the different gene inductions associated with *P. aleophilum*, *P. chlamydospora*, and *P. aleophilum* + *P. chlamydospora*, an induction pattern proper to each treatment appeared. Plant perception of *P. aleophilum* seemed to occur earlier than the perception of *P. chlamydospora* (48 vs. 120 hpi) for nine of the genes out of the eleven that respond to treatments. It is difficult to speculate on the origin of the early response to *P. aleophilum* vs. *P. chlamydospora* because their life traits and the etiology of esca disease is still poorly understood (Bertsch et al., 2013). Although, *P. aleophilum* might develop faster than *P. chlamydospora* at earlier time points, the growth of the two fungi *in planta* is slow and may not differ. Remarkably, the co-inoculation treatment also presented a different pattern, notably for the gene *STS8*. This gene was up-regulated by either *P. chlamydospora* or *P. aleophilum*, but not when *both* species were in the trunk. Note that both species are often isolated together in esca-symptomatic plants. These results indicate that a gene coding a stilbene synthase and involved in synthesizing antifungal compounds in grapevine (Chong et al., 2009) was less expressed when both species were present than when they individually colonized grapevine hosts. This early interaction may also support the hypothesis of a synergistic relationship between *P. chlamydospora* and *P. aleophilum* based on their different enzymatic activities (Valtaud et al., 2009; Luini et al., 2010). However, it remains astonishing that gene expression could differ depending on pathogen inoculation or co-inoculation in grapevine wood. The present results do not provide evidence supporting the synergy hypothesis because quantification of fungal DNA indicates that *P. chlamydospora* was more developed in grapevine wood upon single inoculation compared with co-inoculated grapevine wood, which contained both *P. chlamydospora* and *P. aleophilum*. Conversely, if we consider the expression of the gene *STS8*, both pathogen species may be favored by the smaller IF due to treatment for *P. aleophilum*+ *P. chlamydospora* than for the mock-inoculated wood. This observation, together with the subtle plant perception for selected genes, indicates that this work should be extended to a full transcriptomic study.

A full transcriptomic analysis requires a validation by analyzing the grapevine wood system with RT-qPCR. However, previous studies of grapevine wood are sparse at best (Borges et al., 2014; Yacoub et al., 2016). Taken together, these approaches open a new field in the study of grapevine-microbe interactions in the context of GTDs and open the possibility of analyzing gene expression in trunk tissues. The microarray technique is available and was used to study eutypiosis in leaves (Camps et al., 2010, 2014). The RNAseq technique may highlight changes in gene expression for genes specific to trunk response; a function in trunk tissue that remains to be characterized.

This study also constitutes a first investigation into the possibility of early perception of pathogens by grapevine-trunk tissue, which is why we selected kinetic points from 10 to 120 hpi. Studying later kinetic points would give information about defense, which is of particular interest for future work. We find that a wound masks pathogen perception at 10 and 24 hpi, but pathogen perception of *P. aleophilum* and *P. chlamydospora* diverges at 48 and 120 hpi. Within 6 weeks of inoculation, we should begin monitoring trunk defense to see if a response to esca-associated fungi occurs. The recent work investigating biological control of *P. chlamydospora* by root colonization of *Pythium oligandrum* reports that gene inductions occur within weeks of inoculation in grapevine woody tissues (Yacoub et al., 2016). Defense is induced depending on the biological control agent used for root inoculation, which is consistent with our suggestion of a subtle early perception in grapevine trunk. When combined with investigations into the development of different pathogens in the trunk, we expect future results to clarify how this defense response affects trunk resistance in the context of early interactions with esca-associated fungi.

## Conclusion

The results of this investigation indicate that grapevine trunk perceives fungal pathogens. Nine of the eleven defense-related genes selected in this work indicate that woody tissues perceive whether *P. aleophilum*, *P. chlamydospora*, or both fungi are present in an injury. The results of the present study imply that, despite the important background generated by wounding, gene expression differs depending on whether the wound is inoculated with one or the other of *P. aleophilum* and *P. chlamydospora* or with both *P. aleophilum* and *P. chlamydospora* together. Future full transcriptomic studies should clarify grapevine-microbe interaction in grapevine trunk. Knowledge of such interactions is essential for understanding the molecular mechanisms involved in early colonization of grapevine trunk by esca-associated fungi and for developing alternative tools to control esca-related diseases.

## Author Contributions

RP, JP, SC, and AJ designed the study. RP, JP, CC, and EJ acquired the data. RP, JP, SC, and AJ analyzed the data. RP, JP, SC, and AJ wrote the manuscript. All coauthors discussed and commented the manuscript.

## Conflict of Interest Statement

The authors declare that the research was conducted in the absence of any commercial or financial relationships that could be construed as a potential conflict of interest.

## Supplementary Material

**FIGURE S1 | Local response to internodal wounding in *Vitis vinifera* L. cv. Cabernet Sauvignon clone 15.** The induction factors are relative to the reference gene *EF1-α* and to the gene expression quantified in mock-inoculated plants.
