# Supplementary figures and images for: Variations in Early Response of Grapevine Wood Depending on Wound and Inoculation Combinations with Phaeoacremonium aleophilum and Phaeomoniella chlamydospora
Source: Front Plant Sci. 2016 Mar 11;7:268. doi: 10.3389/fpls.2016.00268 (PMC4786745; doi:10.3389/fpls.2016.00268)

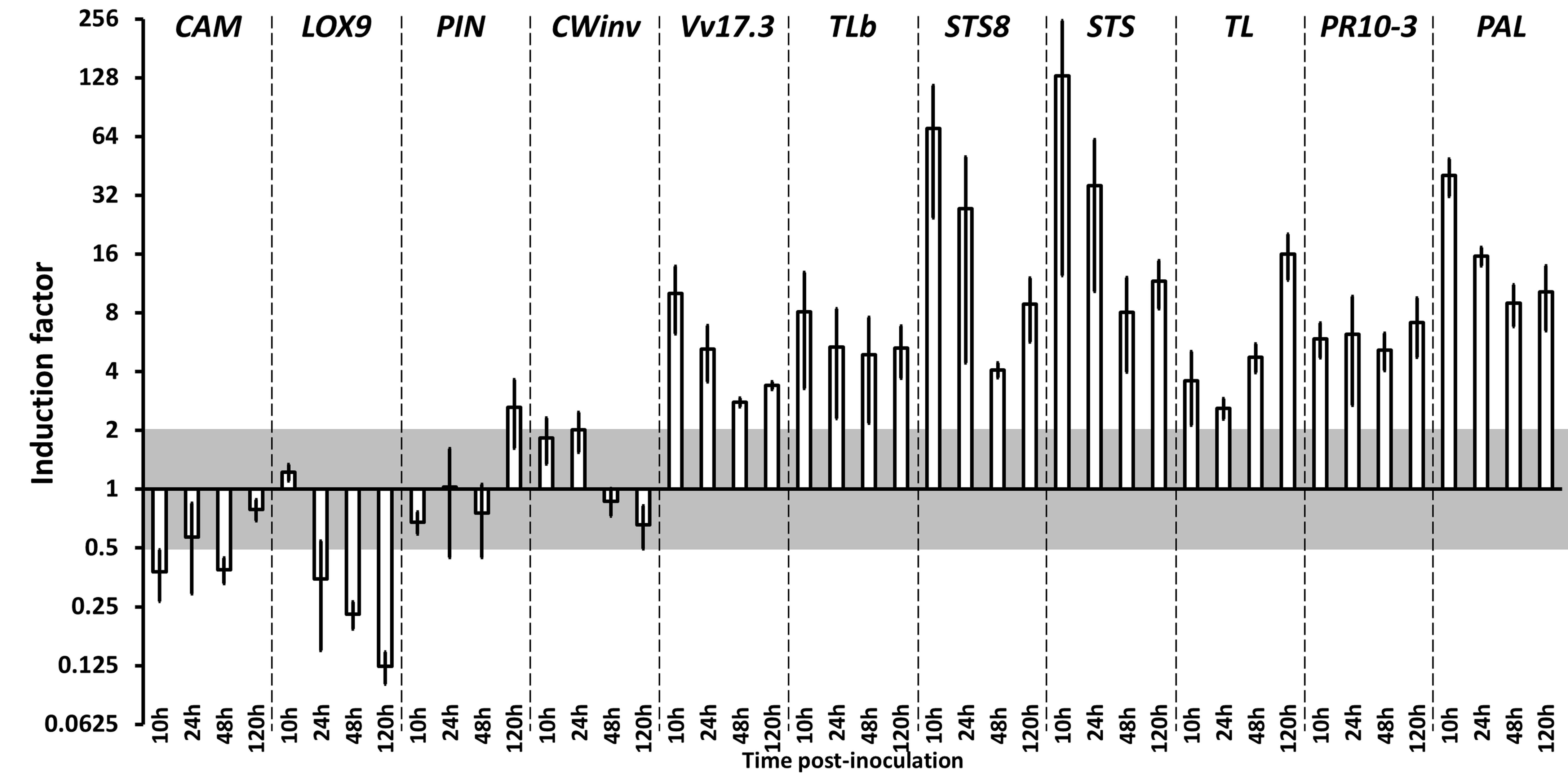

Supplement: Supplementary file 1 [file Image_1.TIF]
